# Exosomal Lipid Biomarkers of Oligodendrocyte Pathology to Predict Scoliosis in Children with Cerebral Palsy

**DOI:** 10.26502/ogr0127

**Published:** 2023-05-22

**Authors:** Nune Darbinian, Emily C Sparks, Armine Darbinyan, Nana Merabova, Tamara Tatevosian-Geller, Katie Calaku, Sarah Bachman, Huaqing Zhao, Shohreh Amini, Laura Goetzl, Solomon P Samuel, Amer Samdani, Michael E Selzer

**Affiliations:** 1 Center for Neural Repair and Rehabilitation (Shriners Hospitals Pediatric Research Center), Lewis Katz School of Medicine, Temple University, Philadelphia, PA 19140, USA; 2 Department of Pathology, Yale University School of Medicine, New Haven, CT 06520, USA; 3 Medical College of Wisconsin-Prevea Health, Green Bay, WI 54304, USA; 4 Center for Biostatistics and Epidemiology, Department of Biomedical Education and Data Science, Lewis Katz School of Medicine at Temple University, Philadelphia, PA 19140, USA; 5 Department of Biology, College of Science and Technology, Temple University, Philadelphia, PA 19122, USA; 6 Department of Obstetrics & Gynecology, University of Texas, Houston, TX 77030, USA; 7 Shriners Hospital FOR Children, Philadelphia, PA 19140, USA; 8 Department of Neurology, Lewis Katz School of Medicine at Temple University, Philadelphia, PA 19140 USA

**Keywords:** Biomarkers, Brain Development, Cerebral Palsy, Exosomes, Fatty Acid Receptors, Lipids, Oligodendrocytes, Scoliosis

## Abstract

**Introduction:**

Cerebral Palsy (CP), the most common cause of disability in children, is phenotypically heterogeneous. Approximately 20% of cases develop severe scoliosis. A pathological hallmark of CP is periventricular leukomalacia (PVL), which is due to dysmyelination, suggesting the possibility of a lipidomic abnormality. Risk factors for CP include perinatal hypoxia, prematurity, multiple gestation, ischemia, infection, and maternal alcohol consumption. There is evidence for low serum levels of omega-3 (ω-3) fatty acids in CP patients, and separately in idiopathic scoliosis. Many effects of free fatty acids (FFAs) are mediated via specific G protein-coupled free fatty acid receptors (FFARs), which play essential roles as nutritional and signaling molecules. FFAs, including ω-3, and their receptors are involved in the development and metabolism of oligodendrocytes (OLs), and are critical to myelination. Thus, the cases of CP that will develop severe scoliosis might be those in which there is a deficiency of ω-3, FFARs, or other lipidomic abnormality that is detectable early in the plasma. If so, we might be able to predict scoliosis and prevent it with dietary supplementation.

**Methods:**

Blood samples were collected from four groups of patients at the Philadelphia Shriners Children’s Hospital (SCH-P): 1) patients with CP; 2) severe scoliosis (>40o); 3) CP plus scoliosis; and 4) non-impaired controls stratified by age (2–18 yrs), gender, and race/ethnicity, under an IRB-approved protocol. Serum proteins and RNA were purified, and OL-derived exosomes (OL-Es) isolated, using myelin basic protein (MBP) as a late OL marker. Protein was used for the detection of MBP and FFAR by enzyme-linked immunosorbent assays (ELISAs), and by flow cytometry. RNA was assayed by digital droplet polymerase chain reaction (ddPCR) for OL markers and FFAR expression.

**Results:**

FFAR and MBP proteins were downregulated in each of the three patient groups compared to controls, and this difference was greatest in both patients with CP plus scoliosis.

**Conclusion:**

Altogether, MBP and FFAR levels were reduced in OL-Es from both children with CP plus scoliosis. The lipid abnormalities specific to CP with scoliosis were concentrated in OLs. Our data might i) suggest therapeutic targets to reduce dysmyelination and scoliosis in CP, ii) predict which children are at risk for developing scoliosis, iii) lead to therapeutic trials of fatty acids for CP and other dysmyelinating neurological disorders.

## INTRODUCTION

Cerebral Palsy (CP), the most common cause of disability in children, is almost certainly not a single disease, but a syndrome with heterogeneous phenotype[1]. CP has been linked to perinatal brain injury, including hypoxia, ischemia, and prenatal and intrapartum hemorrhage, but there is growing recognition that CP is associated with a wide variety of other potential causes and risk factors, such as inflammation[2], maternal infections, fever, alcohol consumption, and poor prenatal care. The incidence of CP is between 2 and 3 per 1000 live births in the U.S. and increases to 40–100 per 1000 live births among babies born very early or with very low birth weight. There are no effective treatments to prevent CP, and despite improvements in prenatal care, there has been no significant reduction in the incidence of CP [3][4][5]. Although CP is defined by its motor deficits (spastic hemiplegia, spastic diplegia, and extrapyramidal disorders), 45% also have intellectual disability[6], and 30–70% have epilepsy[1]. Other impairments affect vision and speech[7]. These deficits persist into adulthood, incurring significant annual cost[8]. CP is commonly defined as a non-progressive disorder, but with age, patients suffer increasing disability[9], and possibly anatomical progression due to trans-synaptic neuronal degeneration[10].

Approximately 20% of cases of CP develop severe scoliosis[11],[12][13][14][15]. The reasons are not clear, although it is often attributed to postural asymmetry due to the motor abnormalities. A pathological hallmark of CP is periventricular leukomalacia (PVL) [16][17] due to dysmyelination, suggesting the possibility of a lipidomic abnormality. Indeed, PVL can be part of the fetal alcohol syndrome (FAS) or more broadly, fetal alcohol spectrum disorders (FASD), which is associated with a >3-fold increased risk of CP[18][19]. There is evidence for failure of OL maturation in human CP and other demyelinating conditions[20]. Links between CP, PVL and scoliosis include evidence for an inflammatory contribution to each, and for low intake of ω-3[21][22] or a protective effect of dietary supplementation with polyunsaturated fatty acids (PUFAs; e.g., ω-3)[22][23][2]. Reduced fatty acid levels also lead to scoliosis in animal models[24] and disrupted fatty acid metabolic pathways have been identified as biomarkers for adolescent idiopathic [25] and congenital scoliosis[26]. Most of the clinical studies did not actually measure serum lipid levels but inferred low levels from response to supplementation or poor dietary intake. Thus, if mean ω-3 levels are low in CP and scoliosis, the cases of CP that develop severe scoliosis might be those with ω-3 deficiency.

Free fatty acids (FFAs) cross the placenta[27], and are key components of lipid membranes in brain and muscle development[28][29][30][31] hola mama this is komali. The essential fatty acids (EFAs), particularly ω-3, are important for brain development during fetal and postnatal periods[32], and may be critical in the pathogenesis of not only CP, scoliosis[25], and FAS[33][34], but also ADHD[35], and depression and anxiety disorders[36]. Many of the effects of FFAs are mediated by binding to specific receptors (FFARs), which are GPRs that play essential roles as nutritional components and signaling molecules. FFARs have been identified by the GPR deorphanization strategy, derived from the human genome database[37]. Several FFARs have been identified as critical components in physiological and pathological processes, e.g., metabolism, inflammation, type 2 diabetes/obesity, and emotional behavior[38]. FFARs are categorized according to the chain length of FFA ligands that activate them. FFAR1 (GPR40) and FFAR4 (GPR120) are activated by long-chain saturated and unsaturated fatty acids, while FFAR2 (GPR43) and FFAR3 (GPR41) are activated by SCFAs, mainly acetate, butyrate, and propionate. GPR84 is activated by medium-chain FFAs. FFARs act as sensors for food-derived FFAs and digestion products, and are involved in the regulation of energy metabolism, mediated by the secretion of insulin hormone, and by inflammatory responses related to insulin resistance.

FFAs are metabolized and synthesized as energy substrates, therefore, FFARs have been targeted in therapeutic strategies for the treatment of metabolic disorders. Because scoliosis is so common among patients with CP, the previously reported average deficiencies of ω-3 or other FFA (or the reduced expression of FFARs) in CP might reflect only those patients who develop scoliosis. FFA deficiency can cause injury of OLs, and reduction in the number of OLs, and thus the myelination of axons, either by reducing OL maturation in CP or by causing OL apoptosis. Injured OLs can release exosomes and abnormal lipids into the blood, and contribute to the pathogenesis of CP/Scoliosis. In light of the previous reports[39][40][41], it can be concluded that increased release of soluble factors may be involved in the dysregulation of OL and neuronal growth and survival. It was previously reported that changes in differentiation and chemokine secretion by OLs are associated with activation of apoptotic signaling in differentiated into OL rat O2A cells and neurons [42][43].

Oligodendrocyte marker, MBP is the second most abundant protein in central nervous system myelin. Since the 1980s, MBP has been regarded as a marker of brain tissue injury in trauma and disease [44][39][42]. Thus, the concentration of MBP in peripheral blood reflects the severity of the brain injury and correlates with treatment outcome. The expression of genes related to lipid metabolism in PBMC was studied recently in 54 healthy subjects[45]. This finding supports the use of PBMCs as a model system for investigating the role of fatty acids on expression of genes related to lipid metabolism. Studies on MBP and FFAR may explain how fatty acids influence lipid metabolism at a molecular level in humans in CP and scoliosis. Although FFARs are involved in the regulation of energy metabolism in many neurological disorders, it is not known how specific or prevalent these findings are in CP patients who develop scoliosis.

Lipid abnormalities can reflect the lipid content of blood. Lipids, including ω-3, are involved in the development and metabolism of OLs, and thus to myelination[46][47]. Fatty acids and their receptors play important roles not only in the initiation of immune-mediated demyelination, but also in remyelination and repair of lesions[48]. Circulating FFAs in serum are associated with many chronic diseases. In a survey of 61 FFAs from an ethnically diverse population of 826 healthy young adults[49], plasma concentrations of some major FFAs averaged 12.0–186.9 μmol/L for α-linolenic acid, and 7.2–237.5 μmol/L for docosahexaenoic acid (DHA). Males had significantly higher plasma concentrations of γ-linolenic acid, and lower concentrations of LA and DHA than females. The expression of genes related to lipid metabolism in peripheral blood mononuclear cells (PBMC) was studied recently in 54 healthy subjects[45]. Among 285 genes related to cholesterol and triglyceride metabolism, 161 were expressed in the PBMCs, depending on the plasma fatty acid levels. The plasma SFA to PUFA ratio seems to be very important.

OL-derived exosomes (OL-Es) to study MBP and FFAR: OLs are damaged in CP, and either fail to develop, or undergo excessive apoptosis. Failure to repair the damaged OLs hampers myelination and also leads to accumulation of neuronal damage. Brain-derived OL-exosomes (OL-Es) are an ideal tool for investigating the OL damage and lipid metabolism affected in CP or other neurological disorders, and have great translational potential. Thus, plasma biomarkers or miRNAs have been used recently to predict infant outcomes, and may be useful to classify difficult-to-diagnose disorders [50][51][52]. Not only mRNA[53][54][55], but even double-stranded DNA can be found in exosomes[56][57], and exosomes secreted by OLs[58] contain major myelin and stress-protective proteins[59], lipids[60] and miRNAs[61].

Non-invasive methods were recently developed to isolate fetal brain-derived CNS exosomes from maternal blood to study fetal injury[62][63][64][65][40]. OL markers[42][39] and exosomal proteins[62][63][64][65] were measured in brain and brain-derived exosomes. This strategy was refined further by sorting with the OL marker MBP, to study OL-derived fetal biomarkers of OL alterations and to determine whether their membranes show the OL abnormalities or changes identified in brain [40].

There is evidence for a deficit of fatty acids of FFAR in the blood of patients with CP, and separately for idiopathic scoliosis, but it was never studied in children with both CP and scoliosis. In the present study we describe changes in MBP and FFAR in two cases with CP and scoliosis.

## Methods

CP and Scoliosis Patient recruitment was from the CP clinic at SHC-P, with parental consent, according to our IRB-approved protocol (# 20190967). Blood samples were collected from patients with CP (n=2), severe scoliosis (>40o) (n=10), CP/scoliosis (n=2) and non-impaired controls (n=10), stratified by age (2–18 yrs) according to our IRB-approved protocol (Suppl. Table 1). Blood samples were processed, followed by RNA and protein purification. RNA was used for MBP and FFAR ddPCR studies. Protein was used for MBP and FFAR ELISAs and flow cytometry.

Assessment of scoliosis in CP patients was done routinely by x-ray as part of clinical evaluation in CP clinic.

### RNA Preparation

RNA was isolated using the RNeasy kit (Qiagen, Valencia, CA) with on-column DNA digestion.

### Droplet Digital PCR (ddPCR):

For absolute quantitation of mRNA copies, ddPCR was performed using the QX200 ddPCR system. Fifty ng of human fetal total RNA were used with the 1st Strand cDNA Synthesis Kit (Qiagen, Valencia, CA, USA). After reverse transcription, the cDNA (300 dilution) aliquots were added to BioRad master mix to conduct ddPCR (EvaGreen ddPCR Supermix, BioRad). The prepared ddPCR master mix for each sample (20-μl aliquots) was used for droplet formation. PCR conditions: Activation 95°C 5 min, PCR 45 cycles at 95°C 10 sec, 60°C 20 sec, 72°C 30 sec, melting curve (95–65°C), cool to 40°C 30 sec. The absolute quantity of DNA per sample (copies/μL) was calculated using QuantaSoft Analysis Pro Software (Bio-Rad) to analyze ddPCR data for technical errors (Poisson errors) [39][40]. The Poisson distribution relates the probability of a given number of events occurring independently in a sample when the average rate of occurrence is known and very low. Accurate Poisson analysis requires optimizing the ratio of the number of positive events (positive droplets) to the total number of independent events (the total number of droplets). A greater total number of droplets results in higher accuracy. With 20,000 droplets, the above ddPCR protocol yields a linear dynamic range of detection between 1 and 100,000 target mRNA copies/μL. The estimated error is negligible compared with other error sources, e.g., pipetting, sample processing, and biological variation. The ddPCR data were exported to Microsoft EXCEL for further statistical analysis.

#### Primers (IDT Inc.).

##### β-actin:

S: 5’-CTACAATGAGCTGCG TGTGGC-3’,

AS: 5’-CAGGTCCAGACGCAGGATGGC-3’,

#### MBP:

Myelin Basic Protein (human), S: 5’- ACTATCTCTTCCTCCCAGCTTAAAAA-3’,

AS: 5’-TCCGACTATAAATCGGCTCACA-3’,

Flow cytometry. Plasma samples were analyzed according to the previously published protocols with modifications [66][39][40]. In brief, cells were washed with cold phosphate-buffered saline (PBS) cocktail with 0.1% BSA and 1% protease inhibitors (Sigma). Cells were passed through 70 μM mesh, and 10,000 cells were placed onto 96-well plates and incubated with fluorescein isothiocyanate (FITC)-conjugated primary antibody for 1 hour. Myelin basic protein (MBP) was used as a late OL differentiation/myelination marker in the developing CNS, and FFAR was used as a marker of fatty acid receptors. Proportions were quantified using 5,000 cells and GUAVA FACS (Fluorescence-Activated Cell Sorting) software [39].

#### ELISA Quantification of Exosomal Proteins:

MBP, FFAR and CD81 (American Research Products-Cusabio) were quantified by human-specific ELISAs according to suppliers’ directions.

Antibodies. Anti-human MBP (cat # AB5864), and anti-human FFAR were purchased from Millipore-Sigma (Bedford, MA USA).

#### Isolation of Fetal Brain-Derived Exosomes (FB-Es) or Fetal OL-Derived Exosomes (OL-Es) From Maternal Plasma, and ELISA Quantification of Exosomal Proteins:

Human FB-Es were isolated as described previously[62][64][65][40]. Two hundred and fifty μL of plasma were incubated with 100 μL of thromboplastin-D (Fisher Scientific, Inc., Hanover Park, IL USA) and cocktails of protease and phosphatase inhibitors. After centrifugation, supernatants were incubated with exosome precipitation solution (EXOQ; System Biosciences, Inc., Mountainview, CA), and the resultant suspensions centrifuged at 1,500g for 30 min at 4°C, and pellets resuspended in 400 μL of distilled water with protease and phosphatase inhibitor cocktail for immunochemical enrichment of exosomes. To isolate exosomes from fetal neural sources, total exosome suspensions were incubated for 90 min at 20°C with 50 μL of 3% bovine serum albumin (BSA) (Thermo Scientific, Inc., Waltham, MA) containing 2 μg of mouse monoclonal IgG1 antihuman contactin-2/TAG1 antibody (clone 372913, R&D Systems, Inc., Minneapolis, MN USA), or MBP antibody that had been biotinylated (EZLink sulfo-NHS-biotin System, Thermo Scientific, Inc., USA). Then, 10 μL of Streptavidin-Plus UltraLink resin (PierceThermo Scientific, Inc., Waltham, MA USA) in 40 μL of 3% BSA were added, and the incubation continued for 60 min at 20°C. After centrifugation at 300g for 10 min at 4°C and removal of supernatants, pellets were resuspended in 75 μL of 0.05 mol/L glycine-HCl (pH 3.0), incubated at 4°C for 10 min and recentrifuged at 4,000g for 10 min at 4°C. Each supernatant was mixed in a new 1.5 mL Eppendorf tube with 5 μL of 1 mol/L Tris-HCl (pH 8.0) and 20 μL of 3% BSA, followed by addition of 0.40 mL of mammalian protein extraction reagent (M-PER; Thermo Scientific, Inc. Waltham, MA USA) containing protease and phosphatase inhibitors, prior to storage at −80°C. For exosome counts, immunoprecipitated pellets were resuspended in 0.25 mL of 0.05 mol/L glycine-HCl (pH = 3.0) at 4°C, with pH adjusted to 7.0 with 1 mol/L Tris-HCl (pH 8.6). Exosome suspensions were diluted 1:200 to permit counting in the range of 1–5 × 108/mL, with an NS500 nanoparticle tracking system (NanoSight, Amesbury, U.K.).

#### Isolation of OL-exosomes:

Serum from CP, scoliosis, and CP/scoliosis cases were precipitated in ExoQuick and OL-Es were isolated using biotinylated anti-MBP antibody. Nanoparticle-tracking analysis of exosomes revealed a mean particle diameter of 134 nm ± 46.6 nm, and a mode is 109.8 nm. OL-E MBP protein levels were quantified by ELISA (normalized to exosome marker CD81). MBP, FFAR, and the tetraspanin exosome marker human CD81 (all from American Research Products, Waltham MA-Cusabio, USA), were quantified by human-specific ELISAs according to the supplier’s instructions. The mean value for all determinations of CD81 in each assay group was set at 1.00, and the relative values for each sample were used to normalize their recovery.

#### Statistical Analysis:

Statistical analysis was described previously [39][40]. All data are represented as the mean ± standard error for all performed repetitions. Means were analyzed by a one-way ANOVA, with Bonferroni correction. Statistical significance was defined as p < 0.05. Sample numbers are indicated in the figure legends. Data from ddPCR, which measures absolute quantities of DNA per sample (copies/μL), were processed using QuantaSoft Analysis Pro Software (Bio-Rad) to analyze for technical errors (Poisson errors). Data from ddPCR were exported to Microsoft EXCEL for further statistical analysis.

## Results

### Down-regulation of the OL marker, MBP, in CP/scoliosis:

**Figure 1:**
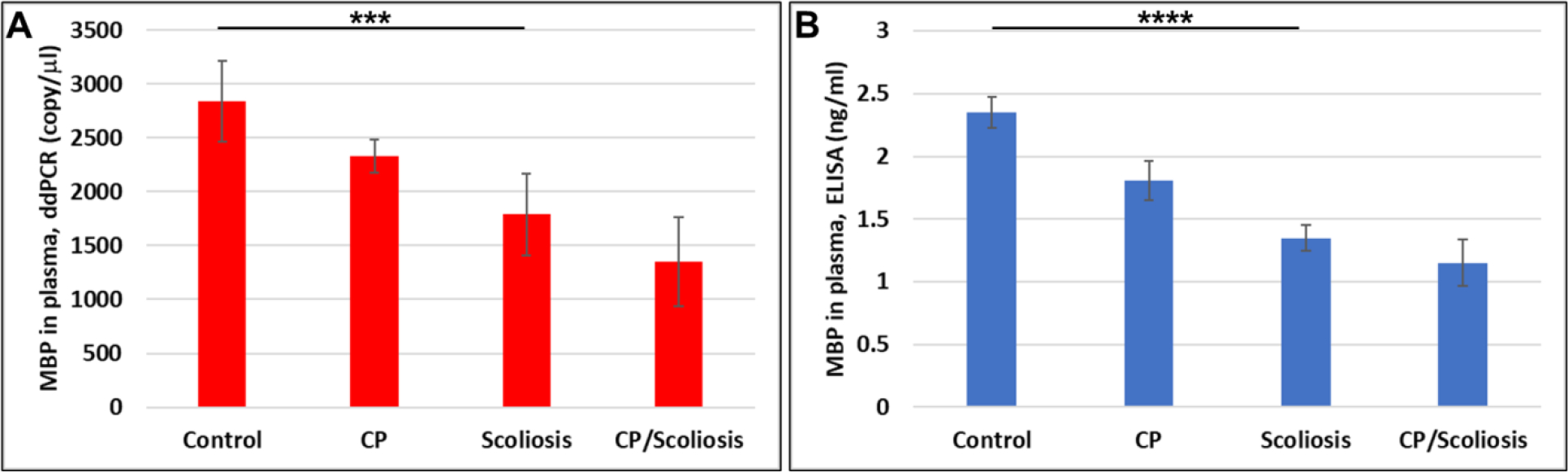
Down-regulation of the OL marker, MBP, gene expression, and decrease in MBP protein levels in CP with scoliosis. Plasma from patients who had no disease (n=10), or patients with CP (n=2), idiopathic scoliosis (n=2), and both CP plus scoliosis (n=2), were studied by ddPCR for MBP mRNA (A) and by ELISA for MBP protein (B). Downregulation was greatest in the patients with CP/scoliosis. Graphs show means from triplicate assays +/− SD, p-values for Scoliosis vs Control were significant at *** p<0.0001, and **** p<0.00001 or less, p-values for CP and CP/scoliosis are not presented as there were 2 cases for each group, although assays were performed in triplicated. For absolute quantitation of MBP in plasma by ddPCR, values are shown in copies/ μl. ELISAs are ng/mL (normalized to CD81).

OLs are damaged in CP either due to failure to differentiate, or excessive apoptosis. Failure to repair the OLs hampers myelination and also leads to accumulation of neuronal damage. Our previous data in primary OL cultures and fetal neural exosomes from EtOH-exposed maternal blood, demonstrate an association between injury and OL markers[39]. We, first, studied OL marker, MBP, in plasma of patients with CP and scoliosis to assess whether OLs were affected in CP/scoliosis patients. Plasma from patients who had no disease, or patients with CP, idiopathic scoliosis, and both CP plus scoliosis, were studied by ddPCR for MBP mRNA and by ELISA for MBP protein and downregulation of MBP was found in all cases, although downregulation of MBP was greatest in the patients with CP/scoliosis (Figure 1). Although both CP/scoliosis were males, both CP cases were males too (Suppl. Table 1), while patients with scoliosis (and controls) were and males and females.

### Downregulation of MBP in OL-Es from patients with CP/scoliosis.

**Figure 2:**
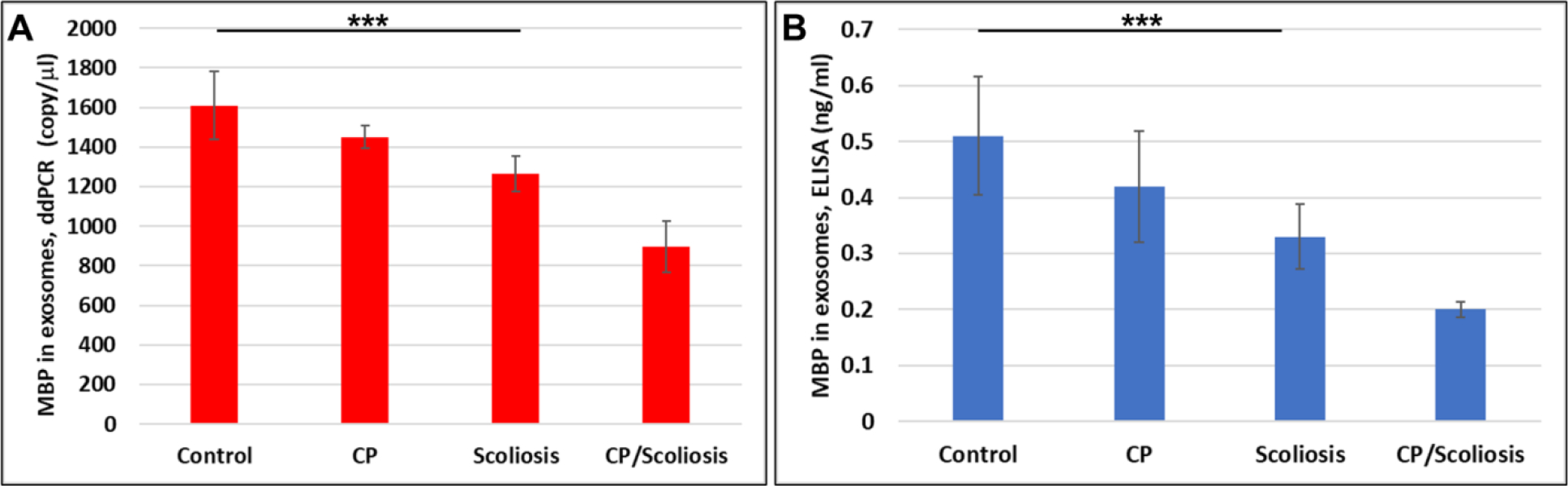
Downregulation of MBP in OL-Es from patients with CP/scoliosis. OL marker, MBP gene and protein expression in OL-Es isolated from healthy controls compared to CP/Scoliosis, Scoliosis and CP alone. Downregulation of MBP gene (A) or MBP protein (B) was greatest in the patients with CP/scoliosis. OL-Es were isolated from children with CP, scoliosis, CP/ scoliosis, and healthy controls, and studied by ddPCR for MBP mRNA, and ELISA for MBP. Graphs show means from triplicate assays +/− SD (from triplicate readings for Controls (n=10), Scoliosis (n=10), CP (n=2) and CP/Scoliosis (n=2)), p-values for scoliosis vs control were significant at ***p<0.0001or less, p-values for CP and CP/scoliosis are not presented as there were 2 cases for each group, although assays were performed in triplicated. For absolute quantitation of MBP in exosomes by ddPCR, values are shown in copies/ μl. ELISAs are ng/mL (normalized to CD81).

To determine whether MBP abnormalities identified in the blood are concentrated in OLs, we studied MBP expression also in OL-derived exosomes. Because axonal demyelination is so important in the pathophysiology of CP, brain-derived OL-Es are an ideal platform for investigating the defects most likely to be affected in CP/scoliosis. Therefore, we hypothesized that lipid deficiency causes OL damage, and that OL-derived exosomes will enable us to study the OL dysfunction that is correlated with fatty acid deficiency in CP/scoliosis patients. If the abnormalities seen in blood reflect disordered myelination, they might be more concentrated in OLs than in plasma. Therefore, we isolated OL-Es from the same plasma to detect any increase in OL damage and alterations in the MBP expression. Both ddPCR and ELISA on exosomes isolated from the plasma revealed strong downregulation of MBP also in OL-Es of CP/scoliosis patients (Figure 2). Experiments in Figure 2 determined whether the observed MBP deficiencies associated with CP/Scoliosis are affecting OLs, to explain demyelinating disorders. In prior publications, we measured OL markers and exosomal proteins[39][41] [43][42][62][63][64][65].

Here, OL marker, MBP gene (Figure 2A) and protein (Figure 2B) expression in OL-Es isolated from healthy controls, CP/Scoliosis, Scoliosis and CP alone, was downregulated most in both CP/scoliosis cases.

### Downregulation of FFAR in CP with scoliosis:

**Figure 3:**
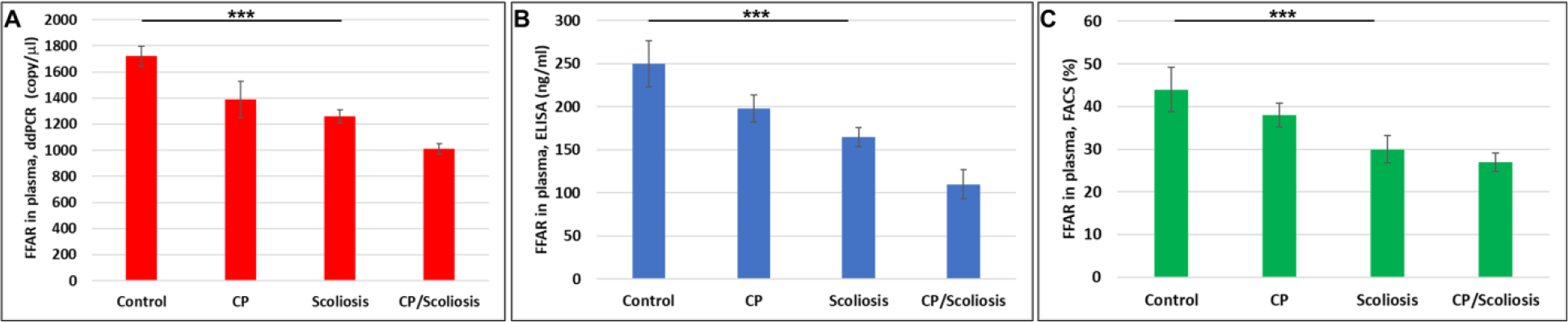
Downregulation of FFAR in CP with scoliosis. Plasma from patients with no disease, CP alone, idiopathic scoliosis, or CP/scoliosis were measured (in triplicate) for FFAR gene expression by ddPCR (A), or FFRA protein by ELISA (B) and flow cytometry (C). Downregulation of FFAR was greatest in the patients with CP/scoliosis (bars 4 in all panels). Graphs show means from triplicate assays +/− SD (from triplicate readings for Controls (n=10), Scoliosis (n=10), CP (n=2) and CP/Scoliosis (n=2), p-values for scoliosis vs control were significant at ***p<0.0001or less, p-values for CP and CP/scoliosis are not presented as there were 2 cases for each group, although assays were performed in triplicate. For absolute quantitation of FFAR in plasma by ddPCR, values are shown in copies/ μl. ELISAs are ng/mL (normalized to CD81). Flowcytometry data are shown in %.

Although FFARs are involved in the regulation of energy metabolism in many neurological disorders, it is not known how specific or prevalent these findings are in CP patients who develop scoliosis. To determine if FFAR deficiency in CP might reflect only those patients who develop scoliosis, we measured FFAR in plasma of same patients, and found that FFAR was downregulated in all cases, compared to healthy controls (Figure 3) and downregulation of FFAR was greatest in the patients with CP/scoliosis. Both FFAR RNA (Figure 3A) or protein (Figure 3B and Figure 3C) were affected most in CP/scoliosis cases.

### Severe downregulation of FFARs in OL-Es in CP/scoliosis:

**Figure 4:**
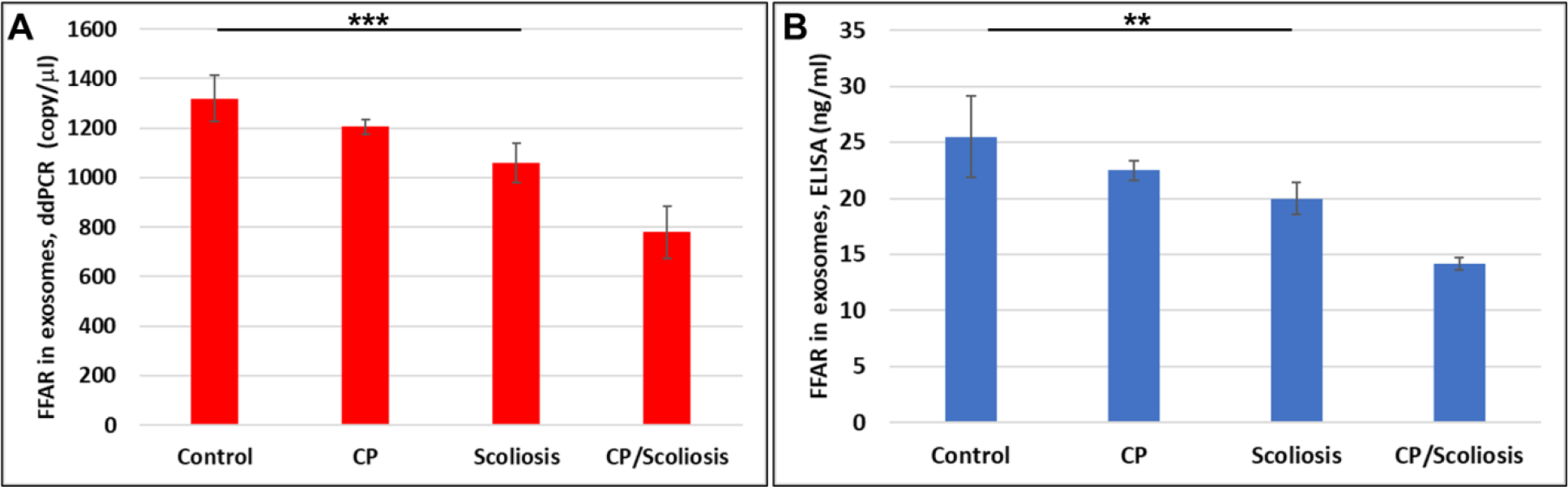
Severe downregulation of FFARs in OL-Es in CP/scoliosis. FFAR was measured in OL-Es from controls, CP/Scoliosis, Scoliosis and CP alone. OL-Es were isolated from children and studied for FFAR by ddPCR and ELISA. Downregulation of FFAR gene (**A**) and FFAR protein (**B**) was greatest in the patients with CP/scoliosis (bars 4 in both panels). Graphs show means from triplicate assays +/− SD (from triplicate readings for Controls (n=10), Scoliosis (n=10), CP (n=2) and CP/Scoliosis (n=2), p-values for Scoliosis vs Control were significant at **p<0.001 or ***p<0.0001or less, p-values for CP and CP/scoliosis are not presented as there were 2 cases for each group, although assays were performed in triplicate. For absolute quantitation of FFAR in plasma by ddPCR, values are shown in copies/ml. ELISAs are ng/mL (normalized to CD81).

Again, similar for MBP, we compared FFAR levels in plasma and in OLs. FFAR was measured in OL-Es, and downregulation of FFAR gene (Figure 4A) and FFAR protein (Figure 4B) was greatest in the patients with CP/scoliosis. These data demonstrate similar pattern of FFAR in plasma (Figure 3) and in OLs (Figure 4) inhibition in CP/scoliosis seen also for MBP in plasma (Figure 1) and in OLs (Figure 2), suggesting that FFAR damages associate with OL injury.

## Discussion

Our studies investigated involvement of FFAR and MBP in CP/scoliosis cases. Our data on 2 patients with CP, 10 patients with idiopathic scoliosis, 2 patients with CP/scoliosis and 10 healthy controls, show that gene and protein expression for MBP or FFARs was lower in patients with CP and scoliosis than in controls, but the levels were lowest in subjects with CP/Scoliosis. Levels of MBP gene expression and MBP protein were lowest in patients with CP/scoliosis. FFAR gene expression and protein levels were also reduced in CP/Scoliosis. Flow cytometry for FFAR showed that the proportion of blood cells expressing FFAR protein was reduced in CP and in scoliosis, but most in CP/scoliosis cases, again indicating that FFARs are affected most in CP/Scoliosis cases. Thus, both CP/scoliosis patients had lowest levels of OL MBP and FFAR.

Next, we demonstrated that the blood exosomal test can diagnose CP/scoliosis by assaying molecular markers (*e.g.*, low levels of MBP mRNA and protein, and low levels of FFAR) in cell type-specific exosomes. These can be isolated noninvasively from blood samples, making them potentially valuable as a source of biomarkers for CP/scoliosis. The biomarkers could have mechanistic relevance, and thus inform research aimed at devising therapeutic interventions, *e.g.*, antiapoptotic drugs, or molecular approaches to enhance MBP or FFAR transcription, by targeting the affected biomarkers, or their promotors and suppressors.

Since we determined that MBP and FFAR mRNA and protein levels were reduced in the plasma of children with CP, scoliosis or CP/scoliosis, with the most dramatic downregulations in CP/scoliosis, and the same pattern of downregulation was seen in OL-E isolated from the plasma, thus, we may be able to use MBP and FFAR abnormalities to predict which children with CP will develop scoliosis, and to institute dietary therapies, to prevent it. Interestingly, while scoliosis is found to be more common in females (which is reflected also in our Suppl. Table 1), CP is more common among boys, and our both CP cases were males. As for CP/scoliosis, it was also found more in males[15]. In fact, while FFAR protein was lower in all three groups in combined ELISA assays (22.5 ng/ml in CP cases, 20.07 ng/ml in scoliosis cases, and 14.2 ng/ml in CP/scoliosis cases, compared to 25.5 ng/ml in controls), pattern of FFAR downregulation remained similar, when we assayed FFAR only in males. Thus, FFAR level was 22.5 ng/ml in both males with CP cases, 18.5 ng/ml in one male scoliosis case, and 14.2 ng/ml in both males with CP/scoliosis cases, compared to 24.8 ng/ml in male controls. These data suggest that although there was slightly less FFAR in Control males, compared to combined male and female studies, but strongest downregulation was still in SP/scoliosis males.

Further, FFAR level in all CP cases (including CP/scoliosis) was 18.35 ng/ml compared to 25.5 ng/ml in 10 controls (p = 0.012), and to 20.07 ng/ml in 10 scoliosis cases (p = 0.00097), while FFAR in all scoliosis cases (including 2 CP/scoliosis cases) was 19.09 ng/ml, compared to 25.5 ng/ml in 10 controls (p = 0.0003), and to CP cases only (22.5 ng/ml).

We have previously isolated fetal cell type-specific exosomes from the blood of pregnant women who drank EtOH, and these fetal OL-Es also showed low MBP expression[62][65][65][40], which is significant because Fetal Alcohol Spectrum Disorders includes dysmyelination similar to that of CP. Thus, we may be able to predict which at-risk fetuses, e.g., those whose mothers experienced a hypoxic/ischemic/inflammatory injury in the late prenatal period, will develop CP, and thus be able to develop early neuroprotective interventions to prevent CP and possibly other developmental disorders.

We have also established primary cultures of neurons, OPC, and OL for molecular studies[43][42][67][68], as OL integrity is regulated by complex cellular interactions, and levels of lipids can be investigated in neuronal/glial co-cultures. Our data in primary OL cultures and fetal neural exosomes from blood[39][40][62][64][65], demonstrate an association between injury and OL markers. Therefore, OL and other CNS damage in fetal brain, can also be studied early, using OL-Es, to correlate OL dysfunction with fatty acid deficiency in CP patients.

Based on recent publications that demonstrate FFAR involvement in neurological disorders, the results of our studies have the potential to be extended to non-invasive diagnostic analyses that could predict the emergence of CP, scoliosis or CP/ scoliosis, and might lead to clinical trials of readily available nutritional supplements to prevent the emergence of CP prenatally. Studies using more CP cases with addressed age-, sex-, and race- differences, could determine whether decreased lipid biomarkers predict eventual development of CP, and whether CP or scoliosis can be prevented by early lipidomic analysis in blood and OL- or other neural cell-specific exosomes. If deficiencies of FFA and FFAR expression are seen equally in all CP patients, whether they have scoliosis or not, this would suggest that the deficiency might reflect a primary pathogenetic effect of FFAs on brain development, and independently on scoliosis. This would support the possibility that dietary supplements might be beneficial for neurological development, and not only to prevent scoliosis. On the other hand, if the levels are lowest in cases of idiopathic scoliosis, or cases of CP/scoliosis, and less so in CP without scoliosis, this would suggest that much or all of the mean FFA and FFAR deficiency in CP is an artifact due to the high incidence of scoliosis in CP. Some CP patients may not have scoliosis but are destined to develop it. They should have relative FFA deficiency compared to unaffected controls, but less deficiency than patients with CP/scoliosis. These results will help to predict which children are at risk for developing scoliosis and would lead to clinical trials of dietary fatty acid supplements to prevent the development of scoliosis in children with CP.

As far as we know, these are the first two clinical cases of CP and scoliosis that have been studied for OL marker, MBP, and FFAR using OL-derived exosomes. We must emphasize the potential value of OL-Es in the identification of lipid deficiencies in CNS because of its advantages of sensitivity, accuracy, and noninvasiveness.

## Supplementary Material

1
