# Supplementary material for: Exosomal Lipid Biomarkers of Oligodendrocyte Pathology to Predict Scoliosis in Children with Cerebral Palsy
Source: Obstet Gynecol Res. Author manuscript; Available in PMC 2023 Aug 3. (PMC10399299; doi:10.26502/ogr0127)
Supplement: 1 [file NIHMS1911459-supplement-1.pdf]

Suppl. Table 1. Clinical characteristics of subjects used in the experiments.

SAMPLE	AGE	GENDER	DX	Race	Height (cm)	Weight (kg)	Scoliosis		Cerebral Palsy
CP/Scoliosis	9	M	CP with scoliosis	white	137	28.3	Diagnosed on 6/1/2010	109 degree curve	diagnosed on 6/1/2010
CP/Scoliosis	12	M	CP with scoliosis	white	157	56.2	Dystrophic scoliosis diagnosed on 12/11/2012	85 degree curve	diagnosed at age unknown
Scoliosis	11	F	Idiopathic scoliosis	white	155	43.7	diagnosed at age 9	53 degree right thoracic and 45 degree Left Lumbar	N/A
Scoliosis	16	F	Idiopathic scoliosis	white	167	54.6	diagnosed at age 3	50 degree right thoracic and 35 degree Left Lumbar	N/A
Scoliosis	15	F	Idiopathic scoliosis	white	160	58.4	diagnosed at age 12	55 degree right thoracolumbar and 33 degree right thoracic	N/A
Scoliosis	15	F	Idiopathic scoliosis	white	153	57.2	diagnosed at age 13	53 degrees right thoracic	N/A
Scoliosis	15	M	Idiopathic scoliosis	white	163	56.3	diagnosed during March 2017	55 degree thoracic and 58 degree Lumbar	N/A
Scoliosis	12	F	Idiopathic scoliosis	white	160.5	39.6	diagnosed at age 11	Right thoracic curve measures 43 degrees and 52 degrees in the left lumbar region	N/A
Scoliosis	16	F	Idiopathic scoliosis	White	161.5	56	diagnosed at age 13	43	N/A
Scoliosis	15	F	Idiopathic scoliosis	White	156	49.5	diagnosed at age 10	55 right thoracic 55 left lumbar	N/A
Scoliosis	11	F	Idiopathic scoliosis	White	143	31.8	diagnosed at age 9	Right thoracic curve measures 55 degrees	N/A
Scoliosis	15	F	Idiopathic scoliosis	White	163.5	68.9	diagnosed at age 12	Right thoracic curve measures 58 degrees	N/A
Cerebral Palsy	14	M	Cerebral Palsy	White	147.3	40.6	N/A		diagnosed at age unknown
Cerebral Palsy	9	M	Cerebral Palsy	White	139		N/A		diagnosed at age unknown
